# What the policy and stewardship landscape of a national health research system looks like in a developing country like Iran: a qualitative study

**DOI:** 10.1186/s12961-022-00905-3

**Published:** 2022-10-28

**Authors:** Atousa Poursheikhali, Mohammed Alkhaldi, Reza Dehnavieh, AliAkbar Haghdoost, Ali Masoud, Somayeh Noorihekmat, Mohammad Reza Cheshmyazdan, Mousa Bamir

**Affiliations:** 1 Health Services Management Research Center, Institute for Futures Studies in Health, Kerman University of Medical Sciences, Medical University Campus, Haft-Bagh Highway, Kerman, Iran; 2 McGill University Health Center, Faculty and Department of Medicine, School of Physical and Occupational Therapy, McGill University, Montreal, Canada; 3 Health System Impact Fellowship, Canadian Institutes of Health Research, Ottawa, Canada; 4 Department of Environmental Health Sciences, Canadian University Dubai, Dubai, United Arab Emirates; 5 Swiss Tropical and Public Health Institute, University of Basel, Basel, Switzerland; 6 Health Foresight and Innovation Research Center, Institute for Futures Studies in Health, Kerman University of Medical Sciences, Kerman, Iran; 7 Faculty of Management and Medical Information Sciences, Kerman University of Medical Sciences, Kerman, Iran; 8 Social Determinant of Health Research Center, Institute for Future Studies in Health, Kerman University of Medical Sciences, Kerman, Iran; 9 Department of Medical Library and Information Science, Kerman University of Medical Sciences, Kerman, Iran

**Keywords:** Health research system (HRS), Health research stewardship, Health research governance, Health research challenges, Iran

## Abstract

**Background:**

The health research system (HRS) is an important national priority that requires a systematic and functional approach. Evaluating the HRS of Iran as a developing country and identifying its challenges reveals the stewardship-related role in how the whole system is operating well. This study aims to assess the HRS in terms of stewardship functions and highlight the enhancement points.

**Methods:**

This study was carried out between March 2020 and April 2021 using a systematic review and meta-synthesis of evidence to examine the Iranian HRS stewardship challenges and interview 32 stakeholders, using a critical case sampling and snowballing approach which included both semi-structured and in-depth interviews. The interviewees were selected based on criteria covering policy-makers, managers, research bodies and nongovernmental organizations (NGOs) in health research-related fields like higher education, research, technology, innovation and science. All data were analysed using content analysis to determine eight main groups of findings under three levels: macro, meso, and micro.

**Results:**

Analysis of the findings identified eight main themes. The most critical challenges were the lack of an integrated leadership model and a shared vision among different HRS stakeholders. Their scope and activities were often contradictory, and their role was not clarified in a predetermined big picture. The other challenges were legislation, priority-setting, monitoring and evaluation, networking, and using evidence as a decision support base.

**Conclusions:**

Stewardship functions are not appropriately performed and are considered the root causes of many other HRS challenges in Iran. Formulating a clear shared vision and a work scope for HRS actors is critical, along with integrating all efforts towards a unified strategy that assists in addressing many challenges of HRS, including developing strategic plans and future-oriented and systematic research, and evaluating performance. Policy-makers and senior managers need to embrace and use evidence, and effective networking and communication mechanisms among stakeholders need to be enhanced. An effective HRS can be achieved by redesigning the processes, regulations and rules to promote transparency and accountability within a well-organized and systematic framework.

**Supplementary Information:**

The online version contains supplementary material available at 10.1186/s12961-022-00905-3.

## Contributions to the literature

- Having a systematic approach to health research helps enhance the research system efficiency, integration and practicality.
- Stewardship needs attention as the essential function of a national health research system (NHRS), especially in a developing country like Iran.
- The stewardship challenges are the root of many other malfunctions of an HRS such as resource management, knowledge management and capacity-building.
- The findings presented herein can serve as the basis for further studies and interventions in improving HRS at the policy-making level.
- A map of an ideal HRS has been imagined to visualize what is needed to achieve a well-organized HRS.

## Background

Health research efforts have received increasing attention globally over the last two decades, and the adoption of systematic approaches to the issue has also been improved [1, 2]. The starting point that drew attention was the gap between health research activities and the highest priorities, discussed at the health research conference in Bangkok in 2000 [2]. Two main concepts are involved: health research and the health research system (HRS). Health research refers to knowledge generation methodology that aims to deal with health problems [3], and it is near the second mode of scientific research. In mode one, science finds its way, but in mode two, it is supposed to benefit the community socially and economically and improve the development path in practice [4]. The second concept is the HRS, also called the national health research system (NHRS) when it applies at the national level. It structures the components and stakeholders active in health research systematically and, more importantly, helps mobilize the resources to address national health needs [5].

The HRS has a critical role in health policy-making by generating evidence [1] of high quality that can be successfully transformed into strategies, norms and policies. As the evidence is of high quality, needs-based and accountable, it also facilitates the path towards universal health coverage [6]. Articulating the health system and its subsystems, based on their functions, helps capture the components, relations and synergies of a systematic framework [7]. Stewardship is an initial function of a health system that allows for meeting the objectives [8]. Stewardship and governance are used interchangeably in HRS frameworks as the primary function of an HRS with mostly the same sub-functions [9–11], as follows: (1) setting vision; (2) developing a national strategic health plan; (3) performance monitoring; (4) setting relations, partnerships, processes, regulations and rules; (5) intelligence generation; (6) ensuring accountability and (7) priority-setting [2, 5, 12–15].

Most of the challenges of the HRSs are rooted in the breakdown of stewardship and governance and related sub-functions (as mentioned above), which is the case for most countries, with more intensity in developing ones [16]. The Iranian HRS is also encountering many malfunctions despite all its improvement in health research over the last three decades [17, 18], so the process of NHRS development has to be redesigned. Most HRS challenges are due to stewardship malfunction, such as a failure of the structural and regulatory framework, processes and monitoring. There are also challenges in setting the research priorities in alignment with national needs. The research bodies are growing, but the outcome is not reflective of the progress, indicating that physical and human development alone is not the answer for national development from a social and human perspective. It can be concluded that other problems need a larger scale of investigation from a stewardship lens. Improving the stewardship helps strengthen the NHRS similarly in Iran and other developing countries [11, 16, 19].

Our research team, consisting of the managers of the health research grant bodies and health-related research centre and researchers, is encountering so many challenges in our daily activities that it motivated us to think about the causes and try to find answers to the questions raised from those challenges. The main question is: how much is Iran's HRS melody harmonious? The first answer that comes to mind, as we have experienced, is not so much! Trying to find the exact and specific causes and solutions based on an initial literature review, the answers were not clear and convergent, so each stakeholder blamed the other. Some blamed researchers for their nonpractical research efforts [20], and others believed that policy-makers are not trusted by the nature of the research and the evidence that can help them assume their roles more efficiently [21]. Meanwhile, others challenge the NHRS and believe that research capabilities are not used as they should be. Reviewing the literature helped us capture the NHRS concept that has received increasing attention globally over the last two decades. It also helps adapt a systematic approach to the issue [1, 2].

### Iranian HRS structure

Iran has a unique health research and education structure due to the integration of health services and medical education [22, 23]. The Ministry of Health and Medical Education (MOHME) is the leading national entity or institution responsible for medical education, health research and technology. Similarly, each university of medical sciences has two vice chancellors for education and research, where research centres are a subset of the vice chancellor for research. However, many other organizations are key stakeholders of HRS regarding its governance and stewardship, directly or indirectly, which include (1) the Islamic Consultative Assembly (Parliament); (2) the Supreme Council of the Cultural Revolution, which defines broad policies in the field of higher education and culture; (3) the Deputy of Research and Technology of the MOHME [7, 18]; and (4) the Plan and Budget Organization [24].

This study aimed to analyse Iran’s HRS challenges regarding the stewardship function. We wanted to enlist the participation of Iranian HRS stakeholders as interviewees across as wide a group as possible and to systematically review the related evidence to ensure that there were no missing data. We expect to provide a base for further studies, implications and reforms. For this purpose, we tried to adopt a practical approach to analysing the data and presenting suggestions. Acknowledging the importance of knowledge translation (KT) in moving towards a qualified HRS, a conceptual drawing of the main recommendations is also shown in Appendices. The picture is developed based on the research findings to illustrate what the Iranian HRS needs in order to be more efficient and improve national health.

## Methods

**Fig. 1:**
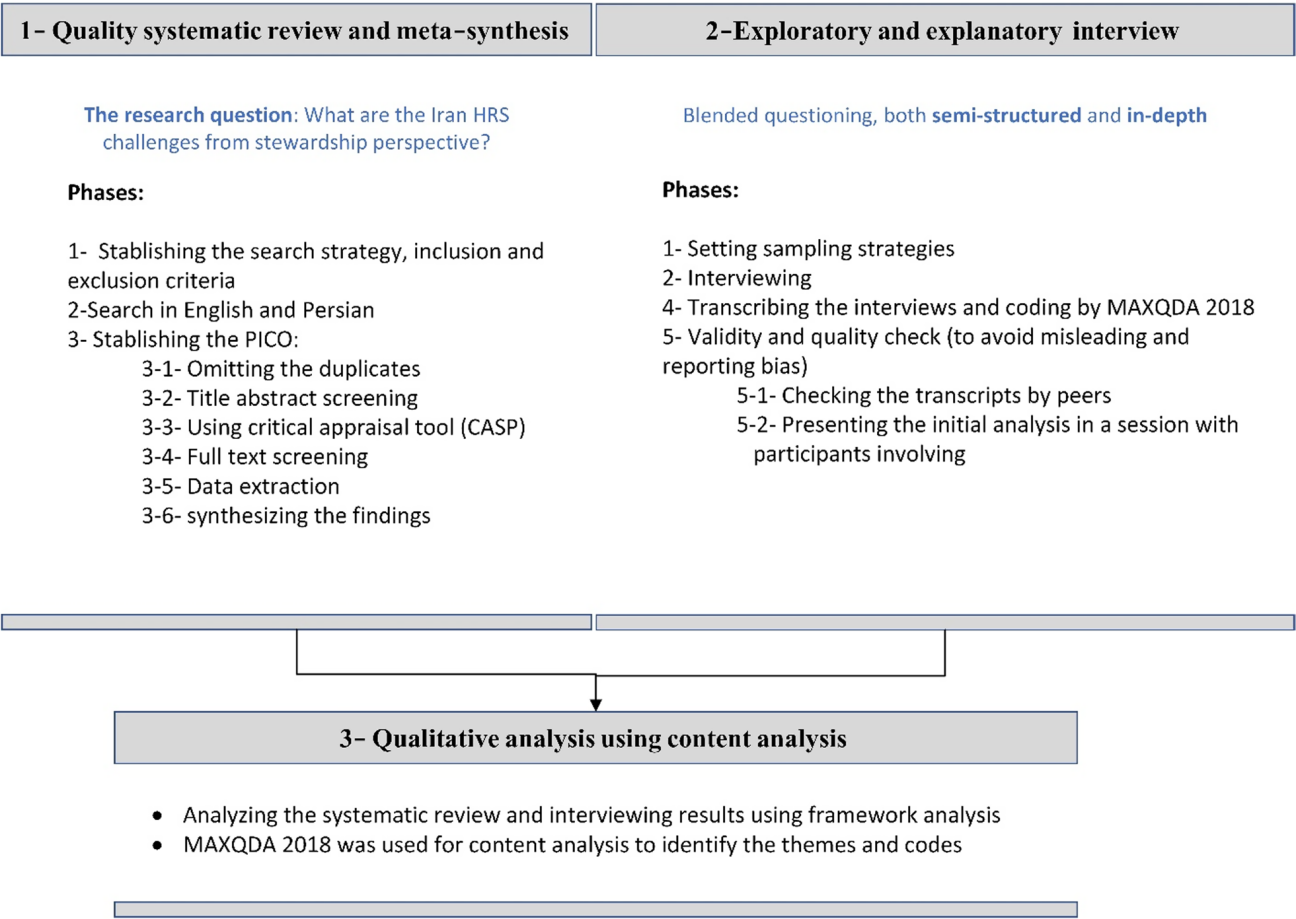
Main phases of the study

This qualitative, cross-sectional, descriptive situation analysis study uses desk review and expert interviews. A brief overview of the phases is presented in Fig. 1. The study was carried out between March 2020 and April 2021.

### Qualitative systematic review

The study asked the question: “What are the challenges of the Iranian HRS from a stewardship perspective?” The systematic review of qualitative studies and a meta-summary and meta-synthesis were performed based on Sandelowski and Barroso’s guidelines [25]. The research team conducted the systematic review with a librarian and data specialist. The Persian databases used in the study were Element, CIVILICA,^1^ Irandoc,^2^ Google Scholar, Scientific Information Database^3^ and the Google local domain. The English databases included Scopus, Web of Science, Science Direct, Emerald, and PubMed. Google was also used to check the grey literature in addition to database coverage in English and Persian. The investigators also asked the experts to introduce any document to help complete the grey literature. The search strategy is presented in Table 3, developed based on SPIDER (Sample, Phenomenon of Interest, Design, Evaluation, Research type) [26].

#### Quality appraisal and data extraction

The Critical Appraisal Skills Programme (CASP) was used for the quality appraisal [27]. The 2020 updated version of the Preferred Reporting Items for Systematic Reviews and Meta-Analyses (PRISMA) is used to report the results, with five steps consisting of omitting the duplicates, title abstract screening, full-text screening, critical appraisal and data extraction [28]. For title and abstract screening, two researchers screened the title and abstract independently based on the inclusion and exclusion criteria and then agreed with a senior researcher to avoid selection bias. The inclusion and exclusion criteria were as follows:

- Inclusion criteria: English or Persian studies with qualitative research design, aimed at exploring:
  - Challenges (Iranian HRS or general research system of Iran) from a stewardship perspective
  - One specific challenge (Iranian HRS or general research system of Iran) related to stewardship sub-functions
- Exclusion criterion: Studies focusing only on research challenges in non-health-related fields.

Critical appraisal was carried out using the CASP tool. Each item was scored as “yes”, “no” or “unclear” depending on their appropriateness, by assigning a score of 1, 0 or 0.5, respectively. As there is no cutoff point for the CASP tool to exclude records, the researchers agreed to exclude those with a score of less than 5 (less than average). Three peers conducted the data extraction phase independently and checked in pairs to ensure data extraction consistency, and papers were selected using a matrix model [29].

The data, including all records from which they were extracted, supporting the conclusions of this article are included within this article in the results section and the discussion (with citations).

An interview approach was employed that included a mixture of semi-structured and in-depth questions, using two types of sampling, critical case and snowball sampling strategies. The interviews were driven by an exploratory approach to capture the challenges in step one. In step two, a group of interviews was carried out using the explanatory method to describe and determine the root causes of the challenges. The interview is reported based on qualitative research reporting standards [30].

#### The sampling strategies for exploratory interviews

The essential HRS subsections and departments (as main stakeholders of the Iranian HRS) were initiated to be the target group of the study. For this purpose, three experts involved in the Iranian health and scientific systems contributed. The selected departments and subsections are government, research centre heads, health innovation system, higher education, science and technology policy-making, senior researchers, HRS, nongovernmental organizations (NGOs), graduate students, and international experts of the Iranian HRS. Participants were identified and selected purposively. After determining the target groups, the strategy was to diversify the participants. The second strategy was to select individual candidates covering more than one category. All the interviewers were asked to propose candidates to be interviewed in parallel with critical case sampling. The interviews continued until they no longer yielded additional information to the data from reviewing the articles.

### Data collection and management

After preparing all expert lists, personal communications with experts were carried out to identify the appropriate time and date for the interviews. One day before the interview, the researcher communicated with experts and sent a summary of the study objectives, consent form and formal invitation. Two methods of structured and in-depth interviews were used for data collection.

The in-depth interview was designed for senior experts and policy-makers to elaborate on more technical and strategic ideas and left it open for them to express their perceptions of Iran’s HRS challenges. The semi-structured interview was used to ask to-the-point questions. The choice of an in-depth approach was based on two main factors:

- Senior stakeholders such as policy-makers with insight about HRS due to their executive and managerial experiences
- Participants whose tendency was to point to the issues without structure and based on their conceptual model.

The researcher had a guide in mind to understand the participant's ideas. The researcher attempted to change the direction during the interview, if necessary. Thus, the in-depth interviews helped develop the conceptual map of the Iranian HRS stewardship challenges. In contrast, the structured interviews added more details and implications of the Iranian HRS stewardship challenges. All the explanatory interviews were conducted using the in-depth approach to capture the roots and causes as much as possible.

The interviews were conducted virtually using Microsoft Team or Skype, and by phone, and the interviewer was in the Kerman province of Iran. The principal investigator conducted the interviews and transcribed the texts. The interviews were recorded with permission using an AnyMP4 screen recorder for the Skype and Microsoft Team interviews and a call recorder for phone calls.

The systematic review and interview results were analysed using the framework analysis method. MAXQDA 2018 software was used for framework analysis. The software helped code the systematic review and qualitative meta-synthesis and interviews. After the framework analysis, all the codes related to the stewardship were reanalysed to develop a conceptual model of the Iranian HRS from the stewardship perspective. The approach was content analysis, and the results of both steps are reported based on consolidated criteria for reporting qualitative research (COREQ) [31].

### Validity and quality check

The following steps were taken to avoid misleading, reporting bias or any other kind of unconscious bias:

The transcribed text of the interviews was checked by a peer in the research team before the qualitative analysis step to ensure the quality and validity of the results.

After the qualitative analysis, a national virtual session was held by the participants involved. The preliminary results of the qualitative analysis of the interviews in alignment with the review results were presented to receive stakeholder feedback and comments. More codes were also mentioned in the session by the participants' debate. The session helped the research team avoid biases, improve the themes and codes, sensitize the stakeholders on results, and let them know about the study progress they have been involved in.

## Results

### Systematic review and meta-synthesis

**Fig. 2:**
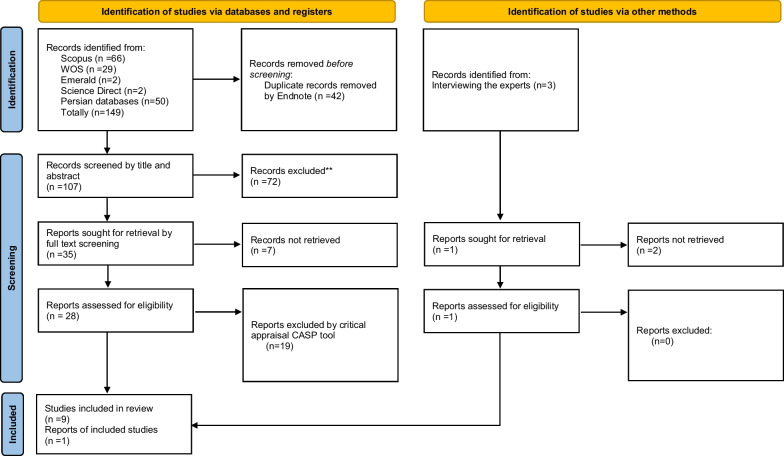
Systematic review PRISMA 2020 flow diagram

The results are sourced from two phases of the systematic review and interviews. After eliminating the duplicates, 149 records were investigated through the selected databases, yielding 42 copies. Three records were also added after asking the experts in the interview phase. After title and abstract screening, 35 records entered the next step, and peers screened 28 full texts to enter the data extraction phase. The other record entered the data extraction by the interview. This record was proposed by senior experts of the Iranian HRS and was not available through database search. Nineteen papers were excluded in the critical appraisal step (their score was less than 5 based on the CASP checklist), and finally, 10 records entered the data extraction. Figure 2 shows the 2020 updated version of the PRISMA flowchart.

### Interviews

A total of 32 interviews were carried out in this study. Twenty-two interviews were structured, and 10 were in-depth. The structured interviews took 45 minutes on average, varying between 30 and 60 minutes, with the in-depth interviews taking 60 minutes on average, and ranging from 45 to 75 minutes. Eleven participants were female and 21 were male; age ranged from 31 to 65 years. Additional information about the participants is presented in Table 4.

The organizations, sectors and individuals identified as the study's target are presented in Table 1. The selected department and subsections are government, research centre heads, health innovation system, higher education, science and technology policy-making, senior researchers, HRS, NGOs, graduate students and international experts of the Iranian HRS. Participants were identified and selected purposively.

**Table 1:** Organizations, sectors and individuals identified to be the study's target (the interview participation scope)

Criterion	Under criterion level 1	Under criterion level 2
Organizations	Ministry of Health	Science, research and technology deputy
		Education deputy
		Research centres
	Medical sciences universities	Science, research and technology deputy
		Education deputy
		Research centres
	Ministry of Science, Research and Technology (MSRT)	Universities
		Research centres
	Entrepreneurship-related organization	
	NGOs	
Sectors	Private sector and health industry	
	National innovation system	
Individuals	International experts who know the Iranian HRS	
	Faculty members and graduate students	
	Top Iranian researchers at the international scale	
	Researchers active in health-related sections	

### Qualitative analysis of the results

The results obtained from both the review and interview are presented after the content analysis. The use of both data collection instruments helped us articulate a map of the challenges and their potential and active relations. The data mentioned here as the study results were obtained from the qualitative analysis of both data sources (desk review and interview) using content analysis to determine the main themes and codes. The results were classified into eight characteristic groups and three levels of macro, meso and micro, combining the content of interviews with the findings from the desk review. The micro level includes research bodies (research centres and universities) and researchers (e.g. faculty members and postgraduate students). The meso level is considered within the MOHME authority, and its education and research deputies are the main structures related to health research. The macro level is considered beyond the MOHME authority. At the national level, the key stakeholders include the Supreme Council of the Cultural Revolution and the Expediency Discernment Council of the System; the MOHME is not the only stakeholder. The results of the review and interviews are presented in Table 5, including quotations and extracted codes.

The eight groups are as follows: (1) leadership; (2) vision; (3) priority-setting; (4) structure; (5) regulation; (6) monitoring and evaluation M&E); (7) communication, networking and collaboration (CNC); and (8) evidence-based/informed decision-making. Table 2 presents a brief overview of the findings. For example, the first feature, leadership, is not clear at the macro level, which means that at this level, there is no transparent, predetermined leadership model or mechanism to determine a shared vision to guide stakeholders. There is a defined structure between the MOHME and its research deputy at the meso level, but the results show malfunctions in leadership. At the micro level, leadership is not functional and efficient based on the results. The status of other features is also presented in Table 2.

**Table 2:** The challenges of the Iranian HRS from a stewardship perspective

Features	Macro At the national scale (not restricted to MOHME)	Meso (Within the authority of MOHME)	Micro Research bodies and researchers
	Status	Status	Status
Leadership	Not clear	Defined structure with malfunctions	Nonfunctional Not efficient
Vision	Not defined	Not defined	Not determined or expressed inefficiently Not aligned with a big picture
Priority-setting	Not well defined Not transparent Not participatory Not systematic Not future-oriented Not efficient	Not well defined Not transparent Not participatory Not systematic Not efficient Not future-oriented Not matched with national development needs	Not aligned with the big picture or not defined
Structure	Not harmonized/multiple actors Overlapped scope of stakeholders Inefficient in conflict of interest management Under the influence of political dominance Not agile	Inefficient Centralized Bureaucratic Under the influence of political dominance Not sustainable Contradictory in conflict of interest management Not agile Not updated	Nonuniform Dependent Gap between theory and practice Inadequate in conflict of interest management Not agile
Regulation	Contradictory Inefficient in: • Incentives • Intellectual property • Capacity-building • Public–private partnerships • Enhancing international scientific relations • Research structure development	Contradictory Inefficient in: • Research process management • Enhancing transparency	Inefficient in: Motivating researchers Performance improvement
Research monitoring and evaluation	Not defined	Not well defined Non harmonized Not standardized Not systematic Not dynamic Inefficient Quantitative One-dimensional	Not well defined Not harmonized Not standardized Not systematic Not dynamic Inefficient Quantitative One-dimensional
Communication, networking and collaboration	Not defined (among critical stakeholders) Not practical	Not well defined (among critical stakeholders) Inefficient Not practical	Not well defined (among research bodies and individuals) Inefficient Not practical
Evidence-based/informed decision-making	Not well defined Not efficient Less trusted Nonfunctional Not a priority	Not well defined Not efficient Less trusted Nonfunctional Not a priority	Not motivating Not efficient in responding

### The macro level

Complexity and lack of collective leadership are the main concerns at the macro level. Multiple stakeholders are not harmonized, with many overlapping and/or contradictions in their scope (regarding health research). This is why CNC mechanisms are limited among key stakeholders of this scale. Concerning the vision as an essential function of HRS stewardship, a lack of shared vision among stakeholders was identified, so despite defined long-term plans at the macro level, leadership and legal obligation to persuade stakeholders of all roles to follow its advice are not embedded in the HRS structure. The priority-setting process is not well defined, transparent, participatory, systematic, future-oriented or efficient. No research M&E system has been defined at the macro level of HRS. The regulations regarding HRS are inefficient regarding incentives, intellectual property, capacity-building and public–private partnership matters, with many contradictions. The development of the research structure has been inefficient. Finally, there is no clear evidence showing a linkage of research to action and policy. Research is not prioritized for potential clients, policy-makers and decision-makers.

### The meso level

The lack of collective leadership was also highlighted at the meso level. The is no defined vision aligned with the macro level. The MOHME authority's priority-setting mechanism has the same malfunction as the macro level, not being well defined, transparent, participatory, systematic, future-oriented and efficient. The priorities of the meso level are not matched with national development needs, so the preferences of the research bodies and researchers are not aligned with upper national ones.

Conflict of interest (COI) management is also important, and the structure of health research is centralized, bureaucratic and influenced by political dominance. The policy dominance also exists at the meso level, leading to the unsustainability of the HRS. The HRS is not aligned with the education system, which is related to the challenge of separating two functions of education and research at both the ministry and its underlying units (e.g. universities and research bodies). Education and research are not the main priority of the MOHME and medical universities [1]. Regulations set by MOHME authority are also contradictory. They are not efficient enough in facilitating and managing the research process (from defining research to evaluation). Laws cannot make the processes transparent, such as setting research priorities and resource allocation. A research M&E system is not well structured, and is not harmonized, standardized, systematic and dynamic. CNC has not been well defined among the critical stakeholders in the authority at the MOHME scale. Finally, evidence-based/informed decision-making also has not entered the HRS structure at the meso level.

### The micro level

The leadership challenges in the previous levels are also transferred to this level. The lack of shared vision among stakeholders results in the misalignment of the health research activities at the micro level with the overall strategic direction of HRS in the country. Furthermore, most research institutions have no vision that manages, regulates or monitors the research. The structure at the micro level is not uniform and varies among cases (in different research bodies). The system of research bodies is dependent on the centralized design at the meso level and is mainly supported by a fixed governmental budget. The design of HRS is not agile enough on all three levels and has not been updated based on new requirements, context and research environment. The rules and regulations are fairly inefficient in motivating research and performance improvement, with challenges in performance M&E at this level. The CNC among research bodies and individual researchers is not well defined, with lower efficiency. The research bodies also could not respond to required evidence with expected quality at the right time, leading to less motivation and trust of both research clients and research bodies.

## Discussion

We conducted a relatively comprehensive study in Iran involving many stakeholders in our in-depth interviews and desk reviews of published and unpublished documents. Like many other developing countries, Iran’s HRS challenges are primarily stewardship and governance [11, 32], even though integrated and coordinated stewardship in any health-related system and subsystem, like HRS, is the starting point for efficient reforms [33]. Iran has started a progressive path in health research, but much work remains to be done. In this study, we tried to adopt a practical approach in determining the issues and recommendations and preparing a base for further studies and interventions. Most importantly, improving the leadership and setting a vision with stakeholder consensus provides the base for other sub-function enhancement like priority-setting. Dealing with the structural and legislative challenges is another aspect. M&E mechanisms and communication and networking among key stakeholders in all three macro, meso and micro levels are also recommended. All thematic challenges make sense in all three groups (macro, meso and micro), but some need special attention in the specific levels, as discussed below.

### HRS challenges at the macro level

The top challenge at this level is national health research leadership. Others are structure, legislation and use of evidence in decision-making. The leadership-related challenges are not limited to HRS or to Iran. *Health leadership* is an international issue [34, 35] in countries of all developmental levels [34–36] and different scales and subsystems of the health sector including hospitals [37], medical education systems [38] and HRS [38–42]. Specifically for HRS in Iran, the leadership challenges are largely due to multiple stakeholders and inconsistent policies [24, 43]. At the same time, leadership is also a matter of future human resources management from different aspects [25, 42–44]. Establishing a focal point to institute the NHRS governance/management is recommended in dealing with such challenges [12, 45–47] so that defining a comprehensive perspective and following strategic plans make sense and work [24, 48].

Next are structural issues: political dominance, COI management, centralization, bureaucracy and lack of sustainability and agility in the HRS. The authority of power and policies is not specific to Iran or any other country, but is a global public health issue [49]. The particular context is also influential in how dominance affects the HRS. Changing the government (by changing the president every 4 years) also changes the HRS in Iran [50–53], making the structure unsustainable. High bureaucracy [41] and centralization of HRS [21, 38, 50] are structural challenges intensified by policy dominance, all of which make the HRS less agile than it should be. COI is a hidden driving force that reduces the research policy connectivity in health systems [54]. The inherently complex nature of health systems makes it challenging to manage the COI in most countries [54, 55]. There was less direct evidence about the status of COI in the Iranian HRS, but it was a concern of policy-makers and research bodies. The main types of COI affecting HRS are policy-maker dual or multiple roles, the financial interest of research bodies, and political interests [56]. The leading solutions are enhancing the regulation and monitoring mechanisms and adopting a proactive approach to managing COI [54, 55].

The third challenge includes laws, legislation and regulations that constitute the initial basis of the health sector and its underlying subsystems on a national and international scale. Legislation and laws related to the Iranian HRS have some malfunctions regarding intellectual property, capacity-building, public–private partnership, research process transparency, motivation and performance improvement. WHO also mentions the restrictive financial and administrative regulations of the Iranian HRS as a primary challenge [41]. One example is intellectual property legislation, which faces some obstacles in Iran. Intellectual property strengthens research findings, commercialization and industry relations [56]. Along with the amendments to the human resources management laws (e.g. performance evaluation, promotions, capacity-building), intellectual property helps solve many HRS challenges including those related to KT, and encourages research bodies towards KT activities [57].

Transparency in research is another issue with some main dimensions including transparency in legislation and performance monitoring, data transparency, open data resources [57], and transparency in analysis and research design. The use of information and communication technology (ICT) enhances access to the legislation related to each step of research process management, which is how legislation is expected to lead to transparency [58]. The current inability of HRS law to enhance public–private partnership [58] and human resources development [59] is another example of the challenges with legislation and laws. By systematically considering these challenges in terms of Iran’s HRS legislation and laws, many contradictions arise mainly due to the multiple trustees in this regard [60].

### HRS challenges at the meso level

At this level, challenges that need to be addressed are priority-setting, M&E and the use of evidence as the basis for decision-making. Priority-setting is not transparent, participatory, systematic or efficient [39, 50]. Reviewing the nine common themes of good health research practices suggests some features for setting priorities. Some consider the context, key stakeholder engagement, determining criteria, implementing an information management system and defining an evaluation mechanism [61]. It is critical to consider a multidisciplinary approach in engaging the stakeholders [62]. Some steps are also proposed internationally by WHO for formulating health research and development priority-setting with similar items. It also recommends developing generic guidance that ensures the flexibility and transparency of the priority-setting process [61]. In Iran, developing regulatory and motivation mechanisms also helps [39]. Last is the incompatibility of the research activities with national needs and priorities [63]. At the same time, it is an indicator used for evaluating the stewardship in an HRS called national focus, which measures the compliance of health research activities with national needs [18].

M&E frameworks aid in the achievement of policy goals and targets. They also make it possible to track stakeholder performance, estimate the effectiveness of the policies and design subsystems. A well-developed M&E framework provides data collection, analysis and sharing [64]. Iran’s HRS faces challenges in its M&E, especially in measuring the performance of the research bodies, evaluating the priority-setting process, being quantitative-oriented and failing to consider the effectiveness of the research. The same is true for the HRS in many other countries in implementing effective M&E, enabling the national HRS to set priorities and develop research policy [65]. Establishing a governance structure in HRS helps mitigate many of these challenges [66], particularly the priority-setting process and facilitating strategic plan development [67]. Besides the M&E challenges, a fixed governmental budget, despite performance, leads to low innovation, competitiveness and motivation in the research environment in Iran [53].‬

The use of evidence in Iran's health policy and decision-making is not well established and not systematic [21, 24]. At the same time, research should be embedded in different phases of policy-making, including identifying and prioritizing issues, developing policy solutions and evaluating the appropriateness of the option. Generally, experts refer to the lack of trust among policy-makers and researchers, the low quality of some research and the weakness of both sides as the primary source of the challenges. The political side of an HRS needs to know how to listen to evidence to enhance the translation of research to action and policy [68] and move towards evidence-informed policy-making. Improving the KT-related knowledge of research bodies and research users can enhance ownership [9]. Other suggestions to improve the evidence uptake are restoring trust between sides, considering the intellectual property and encouraging competition.

### HRS challenges at the micro level

Two issues with more weight at the micro level are promotion law and CNC. The promotion law for faculty members is a clear example of the effects of regulation on capacity-building, performance evaluation and motivation of HRS human resources. It emphasizes quantity instead of research quality and output effectiveness and destroys motivation after receipt of a master's degree [69]. This law does not consider community-based research; it is a prominent trend in the research community worldwide [70]. Unifying the rules in all disciplines and universities, regardless of the requirements of each, is another critical challenge of this law [69]. Alternatively, considering KT activities in the performance evaluation of research bodies and observing intellectual property rights in regulations would work [57]. ‬

The CNC challenges in the Iranian HRS can be classified into three main categories. The first is the CNC of research bodies. There is no networking and data-sharing mechanism among researchers and research centres active in the Iranian HRS, which is the main barrier to knowledge sharing and networking [71]. The second is the CNC between research bodies and the research users, including the policy-makers and the community [24, 39, 71], while networking in HRS is crucial in improving knowledge management [33]. WHO has announced stakeholder engagement as a solution to HRS challenges, especially in developing countries. Malekafzali et al. cited policy-makers' and managers' lack of trust and commitment not to engage the research bodies in related matters. On the other hand, research bodies are not sure whether their comments issue or not [72]. The National Institute of Environmental Health Sciences (NIEHS) endorses six practical community-based participatory research principles: (1) active collaboration and participation at every level, (2) co-learning/fostering, (3) ensuring all research activities are community-based and intervention strategies are culturally adapted, (5) disseminating research staging results properly and (6) defining community as a unit of identity [73]. The third CNC challenge is the lack of CNC among critical stakeholders, with the result that the priority-setting processes lack a systematic framework and transparency [50], primarily rooted in the macro level.

## Conclusion

**Fig. 3.**
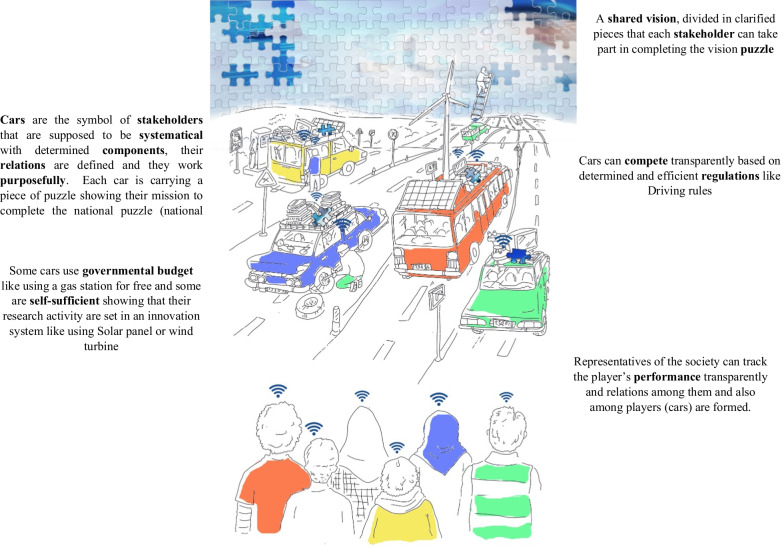
The visualized map of an ideal HRS retrieved from Iran HRS challenges

Adopting a systematic approach to health research activities is binding in some aspects—most notably, establishing vision and targets, determining the components, and defining the relations, processes and rules. Achieving all those features requires effective stewardship of the NHRS. Following up on the roots and causes shows that despite other functional challenges of the Iranian HRS, stewardship issues are more heavily weighted and more fundamental than other HRS weaknesses. The research question stated a challenge that this study investigator encountered every day in their management and research tasks and for which they were trying to find the answers. The Iranian HRS has started its improvement, but there is still work to do. It has missed a potential synergy among critical stakeholders so that improving focal leadership seems essential. This can lead to a vision that mobilizes the research efforts comprehensively and harmoniously, providing a big picture perspective where all resources are mobilized to complete the puzzle. This enhancement will spread to any individual active in HRS as well. Reviewing rules and regulations, strengthening cross-sectoral and intrasectoral links, restructuring the priority-setting, incorporating evidence-based decision-making, increasing transparency and effectiveness in research processes at all three macro, meso and micro levels, and finally strengthening the networking among stakeholders are other solutions that will help enhance the research of the health system from a stewardship perspective. The authors have drawn a conceptual symbolic image of the recommendation to show the systematized recommendations presented in Fig 3.

## Limitations and further studies

Considering the nature of the stewardship scale, we hope to draw the attention of national policy-makers in Iran and related intergovernmental organizations like WHO that can make a difference or at least start the journey to reforms for Iran or any other developing country in improving their HRS through a systematic and framework-based approach. Each sub-function-related recommendation needs attention at the policy-making and leadership level, such as developing practical planning in separate studies. This study had some limitations, such as lack of access to some top policy-makers. If possible, providing a mechanism for the participation of all stakeholder in finding and making sense of the challenges can help improve this study's results and, more importantly, achieve consensus on issues and solutions among stakeholders. This requires the will of top managers and motivation of other stakeholders to participate. We wanted to present the NGO perspective in this study as the community representative, but engaging the community on a broader scale can help as well. As to the methodological aspect, a quantitative analysis assessing the NHRS based on quantitative measures and indexes can complement this study.

### Supplementary Information

**Additional file 1: **Standards for Reporting Qualitative Research (SRQR)a.

**Additional file 2:** Main findings extracted from included studies.

## Data Availability

All data generated or analysed during this study are included in this published article and its Additional file 1 and Additional file 2.

## Appendices

See Tables 3, 4 and 5 and Fig. 3

**Table 3:** Search strategy keywords to identify the challenges of Iranian HRS stewardship based on SPIDER for qualitative studies

Sample			Operator	Phenomenon of interest	Evaluation		
Keywords	Operator	Keywords		Keywords	Operator	Keyword	
“Iran”	AND	“Health Research” OR “Health Research system”	AND	“Governance” OR “Management” OR “Stewardship”	AND	“Challenges” OR “Problems” OR “Weakness” OR “Malfunction”	

**Table 4:** Participant information

Number	Code	Gender	Age	Main sector (selected based on)	Other related sectors
1	GOV_1_	M	51	Government	Futures studies- research and education systems- innovation- science and technology
2	GOV_2_	M	56	Government	Health policy-making
3	GOV_3_	M	47	Government	Research centre head
4	GOV_5_	M	60	Government	Health policy-making
5	GOV_4_	M	62	Government	Health policy-making
6	RCH_3_	F	43	Research centre head	Health innovation system- entrepreneurship
7	HIS_1_	F	47	Health innovation system	Health system management
8	HE_1_	M	65	Higher education	National innovation system
9	STP_1_	M	34	Science and technology policy-maker	Futures studies-senior researcher
10	RCH_1_	F	46	Research centre head	Futures studies
11	SR_1_	M	34	Senior researcher	Faculty member- futures studies
12	ISR_1_	M	35	International senior researcher	Futures studies
13	HRS_4_	M	41	HRS expert	Higher education
14	HRS_1_	F	53	HRS expert	Higher education
15	RCH_4_	M	39	Research centre head	National innovation system
16	RCH_5_	M	37	Research centre head	Medical education
17	RCH_7_	M	48	Research centre head	Health policy
18	RCH_2_	M	51	Research centre head	Health policy
19	RCH_8_	M	40	Research centre head	HRS
20	RCH_6_	M	46	Research centre head	Futures studies in health
21	NIS_1_	M	44	National innovation system	Futures studies- science and technology policy-making
22	NGO_2_	F	36	NGO	Social responsiveness- entrepreneurship
23	NGO_1_	M	39	NGO	Social responsiveness- entrepreneurship
24	PHS_1_	F	35	PhD student	Health policy-making
25	PHS_2_	F	37	PhD student	Health policy-making
26	PHS_3_	M	33	PhD student	Health management
27	Health research_3_	M	35	Health researcher	Futures studies in health
28	Health research_2_	F	31	Health researcher	Futures studies in health
29	Health research_1_	F	35	Health researcher	Futures studies in health
30	HRS_2_	F	59	HRS	Medical education
31	HRS_3_	F	43	HRS	Health research management
32	Int_2_	F	40	WHO Regional Office for the Eastern Mediterranean	Health policy-making

**Table 5:** Review and the interview results

Comparing the results			Interview results example		Systematic review-related references	Code	Analysis level
More details	Not aligned	Aligned	Quotation examples	Affiliation			
-		*	*There is no coherent and sustainable structure that helps in determining a vision, strategy, and program*	Research centre head	Bahadori et al. 2014	Lack of a systematic structure and a national leadership centre for long-term and macro planning	Macro level
-		*	*Our first challenge is the lack of a vision and a big picture to clarify and identify research clusters*	Research centre head	Bahadori et al. 2014 Mansoori 2018	Lack of national vision for HRS	
-		*	*The composition of stakeholders and their involvement in determining research priorities is not practical with a high degree of centralization*	Ministry of Health	Mohammadi and Mesgarpour 2002 Khayatzadeh-Mahani et al. 2013 Badakhshan et al. 2018	Centralization of HRS	
-	–	–	*We are trying to place new ideas in the heart of old and challenging structures, while we do not have the basic infrastructure in some respects*	Researcher	–	Traditional structures with inefficient updates	
-		*	*Who is a medical scientist? Probably a faculty member. What influences their research stance? Helping people? Promotion? Citation? Impact factor? We got metric fixation*	Senior researcher and a faculty member	Tourani et al. 2009 Bahadori et al. 2014 Badakhshan et al. 2018 Karimian et al. 2009	Weak regulatory framework or structure of incentives	
-		*	*Intellectual property laws are a significant obstacle to the commercialization of research achievements. As long as we do not value the idea, we should not expect to move in the direction of promoting innovation*	Health innovation system	Badakhshan et al. 2018	Weak regulatory framework or structure of intellectual property protection	
-		*	*The system is not agile. The process from starting research to ending progress slowly with many formal requirements that are not needed*	Ph.D. student	Bahadori et al. 2014	Weak regulatory framework or structure of research process	
-	–	–	*Laws are weak in supporting students in research activities, especially in determining their research share*	Ph.D. student	–	Weak regulatory framework or structure of capacity-building plan for young researchers	
-	–	–	*If I wanted to outsource the study phases to the private sector, I would face many legal challenges, but I could quickly transfer the budget to the universities for research*	Science and technology policy-maker	Karimian et al. 2009	Weak regulatory framework or structure of Public–private partnerships in research	
-		*	*In general, laws and regulations do not lead to research operations transparency. Financing, human resources development, research centre activities, etc.*	Government	Khayatzadeh-Mahani et al. 2013 Badakhshan et al. 2018	Low transparency of the processes and weakness of regulatory framework in dealing with it	
-	–	–	*We have restricted international relations to the limited exchange of students, and its place in the country’s scientific development is disrupted*	Research centre head	–	Challenge in international scientific relations and weakness of regulatory framework in enhancing it	
It was a challenging code. One of the main actors believes that the Ministry of Health (MOH) is the top leader of the Iranian HRS, and the top-down pyramid is also apparent	*		*There is no horizontal and vertical network of actors active in HRS. There is no institutional mapping. Many parallel workings that lead to waste of resources with low efficiency*	Government	Bahadori et al. 2014 Mohammadi and Mesgarpour 2002 Khayatzadeh-Mahani et al. 2013 Badakhshan et al. 2018 Ramrzani et al. 2018	Non-harmonized multiple actors	Meso level
			*The MOH is the top leader of Iran HRS, and the top-down pyramid is also obvious. So we have the structure, which is not the source of Iran HRS problems*	Higher education top expert			
-		*	*One of our big challenges is that we can't have good management, and as governments change, the presidents of universities change, resulting in a sinusoidal wave in the management of the university that hurts them*	Government	Mohammadi and Mesgarpour 2002 Mansoori 2018	Unsustainable manner of management	
-		*	*We do not have the vision to organize research activities, and we should not expect to meet the needs with this cross-sectional and separate research*	Research centre head	Karimian et al. 2009 Badakhshan et al. 2018	Not placing the research activities on the national research map and with less connection to the national research system	
-		*	*The concepts presented in theory and at the academic level have remained separated and don’t lead to developing measures and interventions to meet the challenges of society or increase welfare and have remained merely at the theoretical level*	Research centre head	Yazdizadeh et al. 2016 Mansoori 2018	The gap between theory and practice	
-	–	–	*I need a laser and an optician in surgery. At the same time, the optician is doing his job in the universities and has never been working with patients and does not know how to work with a laser for humans. Now every specialty is separated. As a result, it prevents the production of technology such as lasers*	Research top manager and entrepreneurship	–	Weaknesses in multidisciplinary and interdisciplinary studies	Micro level
This code was the only one with the slightest consideration for the stakeholders	–	–	*We cannot manage the conflict of interest in HRS. one of the consequences is the biased priorities on health research*	International experts are working both in national and international health research atmosphere	–	Weaknesses in conflict of interest management in HRS	
-		*	*We have summarized the research into the publication of articles, which is the main reason for the decline in the quality of research compared to their main mission, so we have parallel and repetitive work aimed at increasing citations*	HRS top manager	Tourani et al. 2009 Badakhshan et al. 2018 Karimian et al. 2009 Bahadori et al. 2014	Weakness in research monitoring and evaluating	
-		*	*Appropriate networking has not been established between researchers, research organizations, policy-makers, and senior managers* *We have a gap between educational and research structures, both at the university and ministry levels*	Higher education top manager International senior researcher	Khayatzadeh-Mahani et al. 2013 Badakhshan et al. 2018 Ramrzani et al. 2018 Mansoori 2018	Challenges in communications, networking and collaborations, both intersectoral and cross-sectoral	

## Abbreviations

HRS: Health research system
NHRS: National health research system
CNC: Communication, networking and collaboration
M&E: Monitoring and evaluation
MSRT: Ministry of Science, Research and Technology
CASP: Critical Appraisal Skills Programme
MOHME: Ministry of Health and Medical Education

## Footnotes

1 CIVILICA is a knowledge reference private website that indexes and publishes a collection of conference papers and conferences in Iran. CIVILICA is a member of the National Content Consortium and, in this national consortium, is the only provider of data from specialized conferences and seminars in Iran. Available at https://civilica.com/

2 Irandac's core mission is research, science and technology information management, education, research and information collaboration, and science and technology policy support, which is carried out in an organization affiliated with the Ministry of Science, Research and Technology (MSRT) and under the auspices of the Board of Trustees. Available at https://irandoc.ac.ir/

3 The Scientific Information Database (SID) of the Academic Center for Education, Culture and Research Persian is a freely accessible Iranian website for indexing academic journals and access to full text or metadata of academic publishing. Available at https://www.sid.ir/
