# Supplementary material for: What the policy and stewardship landscape of a national health research system looks like in a developing country like Iran: a qualitative study
Source: Health Res Policy Syst. 2022 Oct 28;20:116. doi: 10.1186/s12961-022-00905-3 (PMC9617330; doi:10.1186/s12961-022-00905-3)
Supplement: Supplementary file 1 — Additional file 1: Standards for Reporting Qualitative Research (SRQR)a. [file 12961_2022_905_MOESM1_ESM.docx]

# Standards for Reporting Qualitative Research (SRQR)a

**No. Topic Item**

**Title and abstract**

S1 Title Description of the nature and topic of the study Identifying the study as a qualitative study

S2 Abstract The abstract provides a summary of key elements of the study using the abstract format including background, purpose, methods, results, and conclusions

# Introduction

S3 Problem Formulation We tried to capture the Iran HRS challenges through a systematical lens, focusing on stewardship. Because in the pre-studies we found it as a major function that causes most of the other functions’ problems

S4 Purpose or research question The purpose of the study was to model the stewardship-related challenges of the Iran HRS

# Methods

|  | S5 | Qualitative approach and research paradigm | Qualitative approach and descriptive |
| --- | --- | --- | --- |
|  | S6 | Researcher characteristics and reflexivity | The research question is the research team’s concern and we tried to consider all methodological and technical aspects and be practical as well. The researchers have no bias in the results and the only important thing was to really find the answers |
|  | S7 | Context | Iran HRS has its special and unique structure of higher education and formulates a unique research structure too. We tried to formulate the method and way of analyzing the data considering Iran’s HRS context and structure |
|  | S8 | Sampling strategy | The interviews continued till the saturation and also being sure that we had participants from all stakeholders’ groups |
|  | S9 | Ethical issues pertaining to human subjects | Participants were aware that the session is recorded and the results are presented unnamed |
|  | S10 | Data collection methods | Line 199-220 |
|  | S11 | Data collection instruments and technologies | Line 136-225 |
|  | S12 | Units of study | Number of participants: lines 185-186  Characteristics of participants: lines 188-197  The record entered from the systematic review: Line 174-176 |
|  | S13 | Data processing | Figure 1 shows the main phases of the study. And the processes are presented in lines 140 to 235 |
|  | S14 | Data analysis | line 140 to 235 |
|  | S15 | Techniques to enhance trustworthiness | Peer reviewing in all systematic review steps is also presented on lines 153 to 177.  Also line 227 to 235 presents the considerations for doing the qualitative analysis |
|  |  |  |  |
|  |  | **Results/findings** |  |
|  | S16 | Synthesis and interpretation | The main findings include the development of a conceptual model that has been visualized in appendix 2 |
|  |  |  | , |
|  |  |  |  |
|  | S17 | Links to empirical data | Presented in results description (as additional file) |
|  |  | **Discussion** |  |
|  | S18 | Integration with prior work, implications, | A short summary of main findings; explanation of how findings |
|  |  | transferability, and contribution(s) to the field | and conclusions connect to, support, elaborate on, or challenge |
|  |  |  | conclusions of earlier scholarship in lines 310-431 |
|  | S19 | Limitations | Line 449-458 |
|  | S 20 | Conflict of interest | Line 465-466 |
|  | S 21 | Funding | Line 474-475 |
