# Supplementary material for: What the policy and stewardship landscape of a national health research system looks like in a developing country like Iran: a qualitative study
Source: Health Res Policy Syst. 2022 Oct 28;20:116. doi: 10.1186/s12961-022-00905-3 (PMC9617330; doi:10.1186/s12961-022-00905-3)
Supplement: Supplementary file 2 — Additional file 2: Main findings extracted from included studies. [file 12961_2022_905_MOESM2_ESM.docx]

| **Number** | **Study** | **Title** | **Main findings extracted** |
| --- | --- | --- | --- |
| **1** | Tourani et al.,  2009 | An interview survey on health priority setting practice in Iran | - Insufficient research per capita - Inefficient resource allocation - Political prominence - Weakness of evaluation criteria - Weakness in monitoring - Weakness in measuring effectiveness and costs |
| **2** | Bahadori et al.,  2014 | Challenges of the Health Research System in a Medical Research Institute in Iran: A Qualitative Content Analysis | - Inefficient research process management - Inadequate financing - Lack of monitoring system - Lack of research network - Lack of vision and long-term targets - Political considerations - High bureaucracy |
| **3** | Mohammadi and Mesgarpour  2002 | Systemic approach to health research in Iran and the world | - Centralization of HRS - Lack of evidence based decision making - Unsustainable manner of management - Resource allocation weakness |
| **4** | Yazdizadeh et al.,  2016 | An assessment of health research impact in Iran | - Low impact of the researches - Gap between knowledge creation and knowledge Impact in the Global Innovation Index - Not conducting research based on national needs - The lack of implementation of research results |
| **5** | Khayatzadeh-Mahani et al.,  2013 | Priority setting and implementation in a centralized health system: a case study of Kerman province in Iran | - Non-systematic process of priority setting - little transparency as to how specific priorities are decided - Challenge in implementing the research results - highly centralized system (health priorities are set at the macro level without involving meso or micro local levels or any representative of the public) - lack of collaboration between main bodies and key players - Effect of political pressures on priority setting process - Weaknesses in the implementation of priorities |
| **6** | Badakhshan et al., 2018 | Priority-setting in health research in Iran: a qualitative study on barriers and facilitators | - The rapid turnover of health managers - Scarcity of knowledge about health research priority setting - Centralized decision-making - Integration of heath with medical education - Lack of a national innovation system - Absence of a research map (research puzzles) - Inappropriate leadership of research - lack of transparency in research processes and collaborations - No standardization - Lack of evidence about research gaps - No relation with industries - Inefficiency of faculties’ promotion criteria - Narrow time limit for priority setting - Considering PS as a one-time activity - Ignoring appealing mechanisms of stakeholders - Lack of an efficacious evaluation system |
| **7** | Karimian et al.,  2009 | Investigating the barriers and challenges of research and production of science in medical universities | - Not caring about the private sector - Inefficient evaluation system - lack of a comprehensive database of health research - The university's dependence on a purely government budget - Lack of databases - Lack of coordination of research activities with national development programs - Lack of research-based policy making - Pre-university education challenges - Low independency of researchers and research centers - Effects of political changes like government change - Lack of financial research resources - Weakness of researcher’s competencies and skills - Management related challenges like setting goals and planning - Challenges in doing the original researches - Lack of innovative and competitive researches - Ineffective relation of the policy makers and researchers |
| **8** | Ramrzani et al.,  2018 | Investigate the Barriers and Factors Influencing Inefficiency of Research at Farhangian University: A Grounded Theory Approach | - Financial challenges - Weak collaboration among stakeholders - Inefficient management system |
| **9** | WHO | A Study of National Health Research Systems in Selected Countries of the WHO Eastern Mediterranean Region | - Lack of a well-defined and articulated vision for the Iran national HRS - No defined statement for goals - Lack of broad stakeholder engagement mechanism in HRS - Weakness in partnership amongst national institutions, within the same or with other sectors - No productive mechanism for HRS with the two closely related but much bigger systems, those of higher education and scientific research and technology |
| **10** | Parisa Mansoori 2018 | Evolution of Iran’s health research system over  the past 50 years: a narrative review | - Weakness in use of evidence - a clear articulation of the vision and the goals of health research is absent - too often the policies are not well implemented in Iran - inconsistency in policies; - instability in administration; - limited alignment of policies and available facilities; - lack of communication between researchers and policy-makers; |
